# Draft Genome Sequence of *Pseudoalteromonas* sp. Strain XI10 Isolated from the Brine-Seawater Interface of Erba Deep in the Red Sea

**DOI:** 10.1128/genomeA.00109-16

**Published:** 2016-03-10

**Authors:** Guishan Zhang, Mohamed Fauzi Haroon, Ruifu Zhang, Tyas Hikmawan, Ulrich Stingl

**Affiliations:** a King Abdullah University of Science and Technology (KAUST), Red Sea Research Center, Thuwal, Saudi Arabia; b Ministry of Agriculture, Institute of Agricultural Resources and Regional Planning, Key Laboratory of Microbial Resources Collection and Preservation, Chinese Academy of Agricultural Sciences, Beijing, People’s Republic of China

## Abstract

*Pseudoalteromonas* sp. strain XI10 was isolated from the brine-seawater interface of Erba Deep in the Red Sea, Saudi Arabia. Here, we present the draft genome sequence of strain XI10, a gammaproteobacterium that synthesizes polysaccharides for biofilm formation when grown in liquid culture.

## GENOME ANNOUNCEMENT

*Pseudoalteromonas* (*Gammaproteobacteria*, *Alteromonadales*, *Alteromonadaceae*) was differentiated from the *Alteromonas* genus in 1995, according to the difference in small-subunit rRNA gene sequences (1). *Pseudoalteromonas* is widespread in marine environments and has become an organism of interest in the fields of ecological and pharmaceutical sciences due to its ability to form biofilms and synthesize bioactive molecules (2, 3). To date, 40 *Pseudoalteromonas* genome sequences of different strains have been published, but only three genomes are complete.

Here, we present the genome sequence of *Pseudoalteromonas* sp. strain XI10, which was isolated from the Erba Deep brine-seawater interface (20°43.8′N, 38°11.0′E) in the Red Sea at a depth of 2,400 m. The *in situ* temperature, salinity, oxygen, and pH were measured as 21.0 to 28.0°C, 9.4 to 18.1%, 0.1 to 2.7 mg/l, and 7.6, respectively. The 16S rRNA sequence of strain XI10 showed 99% similarity to that of *Pseudoalteromonas shioyasakiensis* SE3 (4). The strain is nonpigmented and capable of growth at up to 18% (wt/vol) salinity. Similar to other *Pseudoalteromonas* strains, strain XI10 produces large amounts of extracellular polymeric substances (EPS) and forms pellicles and biofilms.

Strain XI10 was grown in Difco marine 2216 enrichment medium with 50% *in situ* brine water at 33°C for 1 week, plated onto Difco marine 2216 agar plates with 10% salinity, and subsequently purified as single colonies. Genomic DNA was extracted from the cultured cells using an alkaline lysis method (5) and subsequently sequenced on the Illumina HiSeq 2000 platform. The raw reads were filtered and trimmed using PRINSEQ (version 0.20.4) (6). SOAP*denovo* (version 1.05) (7, 8), with default parameters, was used to assemble the trimmed reads. The assembly was manually checked and scaffolded based on read mapping. The genome completeness (100%) was assessed using CheckM (version 1.03) (9). Protein-coding open reading frames were predicted by Glimmer (version 3.02) (10). For RNA prediction, rRNAs were predicted by RNAmmer (version 1.2) (11), and tRNAs were predicted by tRNAscan-SE (version 1.21) (12).

The genome of strain XI10, as presented here, is composed of 45 scaffolds, with a total length of 4,537,591 bp (*N*_50_, 400.5 kbp), and contains 3,998 protein-coding genes, 102 tRNAs, and 20 rRNAs. The G+C content of 41.25% is highly similar to that of genomes of other *Pseudoalteromonas* species. Functional annotation by RAST (13) showed the presence of osmoregulation genes related to choline and betaine uptake and saccharide-related genes supporting the adaptation to high salinity and observed EPS production during culturing.

### Nucleotide sequence accession number.

This whole-genome shotgun project has been deposited at DDBJ/EMBL/GenBank under the accession no. LOPY00000000.
